# Bipolar Pharmacotherapy: Lithium from Gold Standard to ‘Commonly Prescribed’

**DOI:** 10.1192/j.eurpsy.2026.10192

**Published:** 2026-07-31

**Authors:** V. Karageorgiou, P. Skapinakis, I. Michopoulos, M. Hadjulis, P. Mitrou, E. Thireos, K. Mathioudakis, R. Gournellis

**Affiliations:** 1 National and Kapodistrian University of Athens, Athens; 2 University of Ioannina, Ioannina; 3 Hellenic Ministry of Health; 4 National Agency for Quality Assurance in Health S.A; 5 IDIKA SA - e-Government Center for Social Security Services, Athens, Greece

## Abstract

**Introduction:**

Lithium remains the most effective prophylactic agent for bipolar disorder (BD), yet reports suggest that clinical practice diverges from guideline recommendations. Understanding the prescribing practices is critical to inform treatment policy.

**Objectives:**

We aimed to characterise prescribing patterns for BD in Greece, focusing on drug category prevalence, adherence, polypharmacy, and diagnostic coding practices.

**Methods:**

We analysed nationwide prescription records for 59,035 BD patients (ICD-10 F30.x, F31.x) covering >1.2 million prescriptions over one year (2020) n the e-Government Center for Social Security (IDIKA) database. The analyses included drug prevalence, medication possession ratio (MPR) to proxy adherence, and a range of subgroup and sensitivity analyses for major psychiatric comorbidities. The derived polypharmacy and MPR phenotypes were statistically modelled with fixed- and mixed-effects models to identify predicting variables.

**Results:**

Sex and age distributions diverged: BD was four times more likely in boys than girls, but prevalence in adults skewed female, peaking at ages 50–59. Lithium was prescribed to 7.6% of BD patients but showed one of the highest adherence scores (median MPR 0.67). Antipsychotics dominated, with 61% of the cohort receiving olanzapine or quetiapine at least once. Polypharmacy occurred in 22% of patients, was as rule short-term, and was strongly predicted by specialist involvement and unspecified severity codes. Mixed-effects modelling showed a substantial influence of unaccounted individual-level factors (R2 61% vs R2 4% in the fixed effects model). In the sensitivity analyses, diagnostic volatility was marked, with 64.4% of patients coded under general F31/31.9, and 37% showing comorbid BD+MDD diagnoses.

**Conclusions:**

In this nationwide cohort study, an underuse of lithium is apparent despite high adherence, and antipsychotics dominate. Polypharmacy may reflect specialist involvement in diagnostically uncertain cases. Previous epidemiological findings of BD sex dimorphism are validated and this is more obvious in older ages, possibly due to more accurate diagnostic processes. This misalignment of guidelines and practice suggests that prescriber habits are partly shaping BD management.

**Disclosure of Interest:**

None Declared

